# Effects of Heterogeneous Diffuse Fibrosis on Arrhythmia Dynamics and Mechanism

**DOI:** 10.1038/srep20835

**Published:** 2016-02-10

**Authors:** Ivan V. Kazbanov, Kirsten H. W. J. ten Tusscher, Alexander V. Panfilov

**Affiliations:** 1 Department of Physics and Astronomy, Ghent University, Ghent, Belgium; 2 Department of Biology, Utrecht University, Utrecht, the Netherlands; 3 Moscow Institute of Physics and Technology (State University), Dolgoprudny, Moscow Region, Russia

## Abstract

Myocardial fibrosis is an important risk factor for cardiac arrhythmias. Previous experimental and numerical studies have shown that the texture and spatial distribution of fibrosis may play an important role in arrhythmia onset. Here, we investigate how spatial heterogeneity of fibrosis affects arrhythmia onset using numerical methods. We generate various tissue textures that differ by the mean amount of fibrosis, the degree of heterogeneity and the characteristic size of heterogeneity. We study the onset of arrhythmias using a burst pacing protocol. We confirm that spatial heterogeneity of fibrosis increases the probability of arrhythmia induction. This effect is more pronounced with the increase of both the spatial size and the degree of heterogeneity. The induced arrhythmias have a regular structure with the period being mostly determined by the maximal local fibrosis level. We perform ablations of the induced fibrillatory patterns to classify their type. We show that in fibrotic tissue fibrillation is usually of the mother rotor type but becomes of the multiple wavelet type with increase in tissue size. Overall, we conclude that the most important factor determining the formation and dynamics of arrhythmia in heterogeneous fibrotic tissue is the value of maximal local fibrosis.

Cardiac arrhythmias remain one of the largest causes of death in the industrialised world. In many cases, cardiac arrhythmias occur in a structurally abnormal heart where the properties of cardiac tissue have changed. The problem of substrate, that is, of identification of properties of cardiac tissue that predispose to cardiac arrhythmias, is of great interest. Structural changes of cardiac tissue, also called remodelling, occur as a result of many cardiac diseases. For example, in the ventricles of the heart, remodelling occurs after myocardial infarction and during heart failure^1^,^2^. In atria, it occurs during sustained atrial fibrillation^2^,^3^ and under many other pathological conditions. Typically, tissue remodelling has several components: change in the properties of ionic channels (ionic remodelling), a reduced number of gap junctions, changes in cell size, and changes in overall tissue structure. The latter mainly occurs as a result of the proliferation of non-excitable cardiac fibroblasts and an increase in the collagen fibres secreted by these fibroblasts, together called fibrosis. Interstitial fibrosis accompanies tissue remodelling in most of the aforementioned cases, and the percentage of fibrotic tissue can increase up to 40%^4^. As the presence of non-excitable cells substantially affects cardiac wave propagation, it is not surprising that fibrosis affects the onset of cardiac arrhythmias and is considered an arrhythmogenic condition^5^.

The relation of fibrosis and arrhythmias has been investigated in several experimental studies. It has been shown that the inducibility of ventricular arrhythmias increases in a nearly linear fashion with the amount of fibrosis^6^. In addition, studies^4^,^7^,^8^ have shown that the structure and spatial distribution of fibrosis play a crucial role in arrhythmogenesis. However, as discussed above, tissue remodelling affects many properties of cardiac tissue simultaneously. Indeed, the importance of heterogeneity in distribution of other factors involved in excitation wave propagation has also been demonstrated experimentally. For example, De Bakker^9^ showed that in human and in mice hearts, the onset of cardiac arrhythmias is associated with heterogeneity in Cx43 distribution rather than its mean levels. In addition, it was shown that remodelling induced decreases in *I*_Na_^10^ play an important role in the onset of arrhythmias. Since many factors involved in tissue remodelling appear to have an arrhythmogenic effect, it is hard to experimentally determine the exact role of fibrotic tissue in arrhythmogenesis. Furthermore, the spatial variation of fibrosis is difficult to control in experiments. Therefore, alternative approaches are needed to determine the importance of heterogeneity in fibrotic patterns on arrhythmia onset. Mathematical modelling of cardiac excitation is a particularly suitable approach since it allows one to study fibrosis independently from the other remodelling factors, to precisely control levels and patterns of fibrosis, and study a large number of different fibrosis patterns.

Mathematical modelling has been widely used to study the effects of fibrosis on cardiac arrhythmias. In a series of papers^11^,^12^,^13^,^14^, the effect of diffuse fibrosis on the onset of arrhythmias due to steep APD restitution was studied. This research led to the paradoxical finding that while diffuse fibrosis promotes spiral wave formation, it suppresses spiral wave fragmentation. It was shown that fibrosis reduces the possibility of steep-restitution induced spiral breakup due to an increase in the period of spiral wave rotation. Furthermore, it was shown that this period increase can result in the onset of 3D instabilities, such as negative filament tension^15^. Thus, while diffuse fibrosis suppresses steep restitution induced spiral breakup it promotes alternative 3D spiral breakup mechanisms^16^.

In addition, a lot of research has focused on the effect of possible myocyte-fibroblast coupling on the conduction velocity and spiral wave dynamics^17^,^18^. The relation of fibrosis and extracellular electrograms was studied in^19^ in a model of human atrial tissue. It was shown that the presence of fibrotic tissue in the atria may be responsible for complex fractionated electrograms. Furthermore, it was demonstrated that ablation of the fibrotic areas producing the fractionated electrograms may terminate atrial fibrillation. The role of fibrosis in the initiation and maintenance of atrial fibrillation was also demonstrated in an anatomically accurate setup using detailed fibrosis models in^20^,^21^,^22^.

Although some of these computational studies have investigated the effect of different types of fibrosis on wave propagation (e.g.^19^), so far no systematic study has been performed on the effects of the heterogeneity of fibrosis on wave propagation and arrhythmia generation. In addition, none of the aforementioned studies have targeted the question regarding the type of fibrillation occurring in fibrotic tissue (whether it is of a multiple wavelet or of the mother rotor type) and how this depends on the properties of the cardiac tissue.

In this paper, we use a human ventricular cardiomyocyte model to study the effect of spatial heterogeneity of diffuse fibrosis on the onset of cardiac arrhythmias. For that, we developed a generic spatial model of fibrosis, which allowed us to change the degree of heterogeneity of fibrosis (amount of difference in local fibrosis levels) and the characteristic size of the heterogeneity (size of tissue patches with a constant fibrosis level). Our study focuses on the question whether heterogeneity in the distribution of fibrosis is an important additional factor contributing to the onset of cardiac arrhythmia. In addition, we aim to answer how this depends on both the degree and the characteristic size of the heterogeneity. Finally we study how heterogeneous fibrosis affects spatiotemporal arrhythmia dynamics and characterise whether fibrillation is of the mother rotor or of the multiple wavelet type, and how this may depend on tissue characteristics.

## Methods

### Mathematical model

We used the ten Tusscher and Panfilov model^23^,^24^ for simulating the behaviour of a ventricular cardiomyocyte:


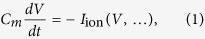


where *V* is the transmembrane voltage for the cardiomyocyte, *C*_*m*_ is the membrane capacitance, and *I*_ion_ is the sum of all ionic currents, which depends on *V*, on the gating variables, and on the concentrations of intracellular calcium. We used the set of parameters that corresponds to a slope of 1.1 of the restitution curve as described in^23^,^24^.

Fibroblasts were assumed to be inexcitable and electrically disconnected from the myocytes, causing them to be passive obstacles for wave propagation.

For our spatial model of a 2D cardiac tissue with fibrosis, we used a rectangular grid with a size of 512 × 512 nodes, where each node can be occupied by either a cardiomyocyte or a fibroblast. Thus, we assume that the area occupied by a cardiomyocyte is the same as the area occupied by a fibroblast—a square with the size of 250 *μ*m.

Following these assumptions the spatial model can be described as:


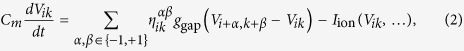


where (*i*, *k*) is a position of a node occupied by a cardiomyocyte (because of the non-conducting character of fibroblast, computation of voltage is not needed in points occupied by fibroblasts), *g*_gap_ is the conductance of the gap junction channels that couple two neighbouring myocytes, and 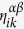 is the connectivity tensor that describes the presence of electrical coupling between neighbouring myocytes and the absence of coupling between myocytes and fibroblasts:


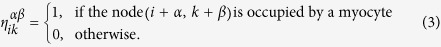


Conductance of the gap junctions *g*_gap_ was taken to be 103.6 nS, which results in a maximum velocity planar wave propagation in the absence of fibrotic tissue of 72 cm/s at a stimulation frequency of 1 Hz, in agreement with clinical data^25^.

### Fibrosis distribution

To generate a uniform fibrosis distribution with a mean level of fibrosis of *f*, we set each node of the grid to be either a fibroblast with the probability *f* or a myocyte with the probability (100% − *f*).

For generating a heterogeneous distribution of fibrotic tissue, we used two extra parameters: the extent of heterogeneity *σ* and the spatial size of heterogeneity *l*. We divided the 2D tissue into squares of the size *l* × *l*. For each square, we assigned a certain local fibrosis level. We used 4 possible values to assign local fibrosis levels:


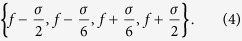


Thus, the meaning of *σ* is the difference between the maximal and the minimal possible local fibrosis levels. After assigning the local fibrosis level *f*_loc_ for a square, we set each node within that square to be a fibroblast with the probability *f*_loc_ or a myocyte with the probability (100% − *f*_loc_).

As one can see, our choice of representing the degree of heterogeneity *σ* implies the following restriction:


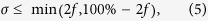


since the local fibrosis level can be neither smaller than 0% nor larger than 100%.

### ECG computation

The (pseudo-)ECG was computed using the following formula:


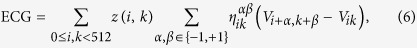


where the transfer function 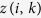 was determined by:


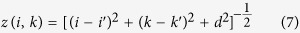


and the measuring point 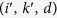 was positioned at (256, 256, 100), that is, at a distance of 2.5 cm along the *z*-axis above the centre of the 2D tissue.

### Ablation modelling

The (pseudo-)ablations were modelled by changing the connectivity tensor *η*. If a node occupied by a cardiomyocyte underwent an ablation procedure, the cardiomyocyte was replaced by an empty node, and the connectivity tensor *η* was recomputed in accordance with (3).

### Implementation

The system of coupled ordinary differential equations (2) was solved by the forward Euler integration method, using the timestep of 0.02 ms, similar to previous studies^23^,^24^. The equations for gating variables were integrated using the Rush-Larsen algorithm.

The numerical solver was implemented with the C and C++ programming languages, using the CUDA toolkit for performing the majority of computations on graphical processing units. The auxiliary tools for manipulating the connectivity tensor, visualisation, and computing the ECG were implemented using the OCaml programming language. Computations were performed with single precision and run on an Intel Core i7-3930K machine with two GeForce GTX 780 Ti graphics cards.

The random number generator was taken from^26^. The seed for the random number generator was taken independently for each simulation.

## Results

### Heterogeneous vs homogeneous fibrosis

**Figure 1:**
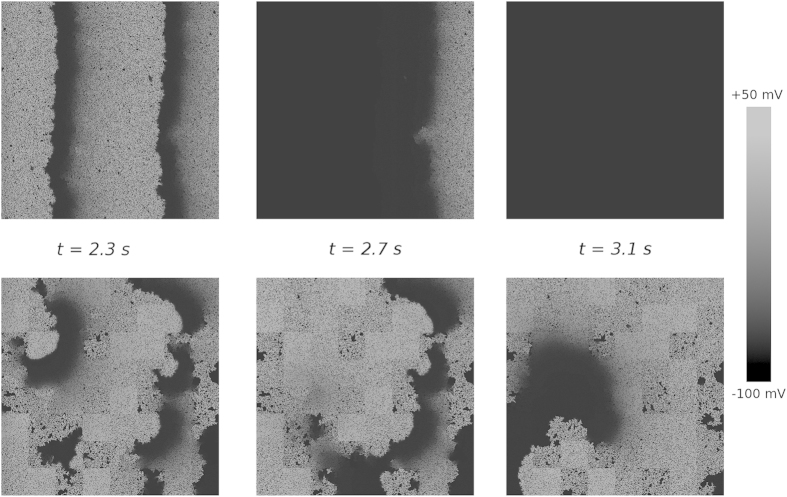
Induction of reentry by burst pacing. The electrical activity is given for the moment when the last stimulus is delivered (*t* = 2.3 s), for 0.4 s after that, and for 0.8 s after that. The shade of grey shows the transmembrane voltage. Top row: homogeneous fibrosis of 25%; bottom row: the same amount of mean fibrosis distributed heterogeneously (*σ* = 25%, *l* = 16 mm). Left column: the state of the medium after delivery of the last stimulus. Middle column: the state of the medium when the last pulse leaves it. Right column: the activation pattern established after termination of stimulation.

We studied the induction of arrhythmias by high frequency pacing for homogeneous and heterogeneous distributions of diffuse fibrosis. We applied a burst pacing protocol of 10 stimuli with a period of 240 ms and monitored the induced activity. Figure 1 shows the results of typical simulations. The top row corresponds to a homogeneous fibrosis distribution with a mean level of fibrosis *f* = 25%. The bottom row shows the results for a heterogeneous distribution of fibrosis with the same mean level *f*, an extent of heterogeneity of *σ* = 25% and a heterogeneity size *l* = 16 mm. For the homogeneous distribution, the electrical activity vanished with the termination of pacing (shown in the top right part of Fig. 1). However, for the heterogeneous distribution, we observed sustained electrical activity.

To study the effects of mean fibrosis level (*f*), extent of heterogeneity and (*σ*) and spatial size of heterogeneity (*l*) we performed a large series of simulations for different combinations of parameter values (*f*, *σ*, *l*). However, we observed that the outcome of our simulations, whether or not sustained electrical activity occurs, was highly stochastic in nature. The Results depended not only on the mean value of fibrosis and the pattern of heterogeneity, but also on the specific locations of randomly distributed fibrotic cells. Because of this stochasticity, we generated for each specific (*f*, *σ*, *l*) parameter setting 50–100 different fibrosis patterns, by using different seeds for the random number generator before creating the pattern. We subsequently performed a statistical analysis of our simulation results, determining the boundary between parameter settings for which at least 25% of simulations results in persistent spiral wave activity and parameter settings for which fewer simulations result in persistent electrical activity.

**Figure 2:**
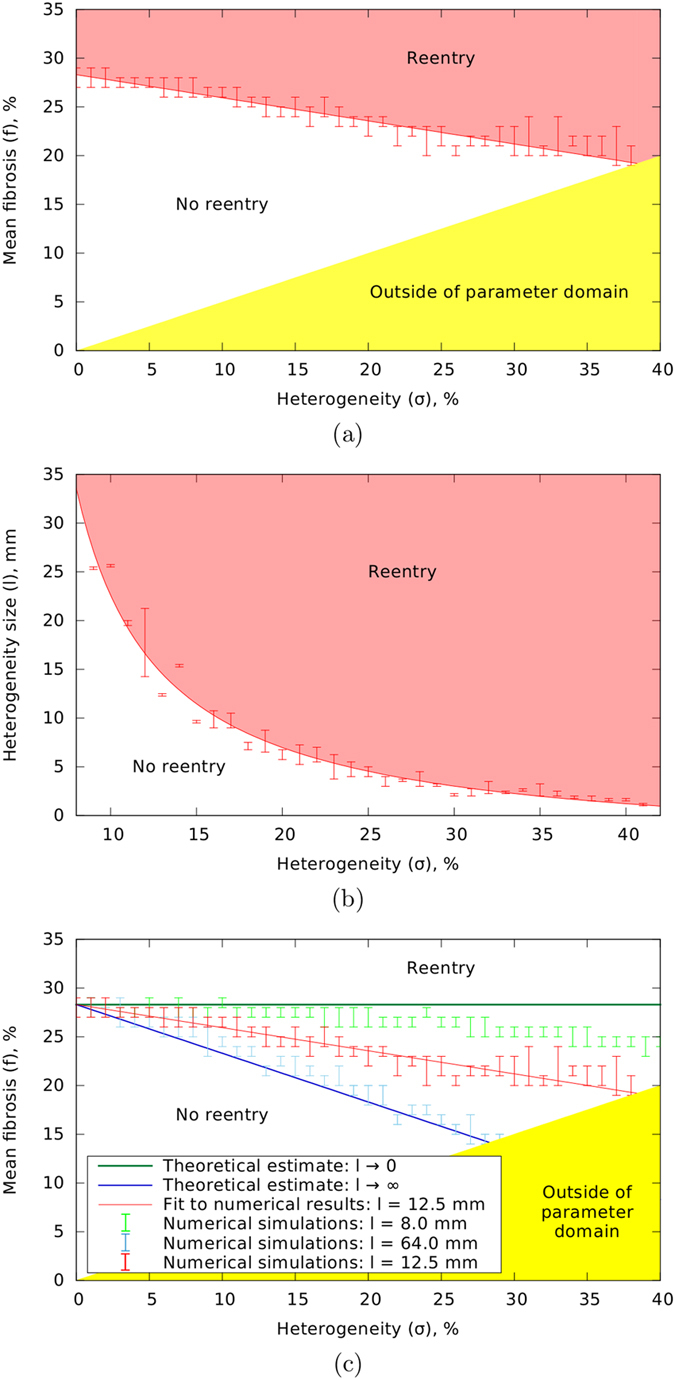
Effect of the mean fibrosis *f*, the width of fibrosis distribution *σ*, and the size of heterogeneity *l* on the probability of reentry generation: (**a**) phase diagram for *σ* and *f* when *l* = 12.5 mm; (**b**) phase diagram for *σ* and *l* when *f* = 25%; (**c**) the same as (**a**) but also includes different values of *l*. In the regions labelled “reentry” the induced activity occurs in more than 25% of cases. The dark green and the dark blue lines in (**c**) are theoretical estimations for the boundary for very small or very large *l* respectively. The light green and the light blue points in (**c**) are the results of simulations for *l* = 8.0 mm and *l* = 64.0 mm respectively. The red lines are the fits to the simulation data. The yellow region shows undefined parameter combinations where *σ* < 2*f* (see Methods).

In the top panel of Fig. 2, we show two phase diagrams illustrating the effect of heterogeneity of fibrosis on reentry generation. In each of the diagrams, there are two regions marked with “reentry” and “no reentry” which correspond to parameter settings for which the probability of inducing reentrant activity is higher or lower than 25% respectively. In Fig. 2a, this phase diagram is shown for the *σ*—*f* parameter space when *l* = 12.5 mm. We can see that reentrant activity occurs for homogeneous fibrosis (*σ* = 0) if the mean fibrosis level *f* ≈ 28% and higher. With the increase of heterogeneity *σ*, the minimal value of the mean fibrosis level *f* required for reentry formation decreases linearly. In Fig. 2b, a similar phase diagram is given in the *σ*—*l* parameter space with fixed *f* = 25%. We see that reentrant activity is more difficult to trigger for smaller values of the size of heterogeneity *l*. The boundary separating the two regions has a hyperbolic shape.

To further investigate the effect of heterogeneity size *l* on reentry generation, we calculated phase diagrams in the *σ*—*f* parameter space for two additional values of *l*. Figure 2c shows the results of these simulations. We see that for a smaller size of *l*, the boundary becomes less steep, whereas for a larger *l*, the slope becomes steeper (more negative). We can interpret these results as follows. If we consider the case of a very small value of *l* the difference between heterogeneous and homogeneous fibrosis disappears due to spatial averaging. Therefore, we expect that the probability of arrhythmia induction for this case is the same as for the case of homogeneous fibrosis and does not depend on the extent of heterogeneity *σ*. This corresponds to the horizontal green line in Fig. 2c. We indeed see that the boundary computed for *l* = 8 mm (light green points) approaches that line. This indicates that heterogeneity at a small spatial scale has much less influence on the probability of spiral wave formation.

On the other hand, if we consider the case of a relatively large *l*, then the patches of fibrosis can be considered independent, in the sense that each of them can be considered as a region with homogeneous fibrosis. The value of such local fibrosis ranges from (*f* − *σ*/2) to (*f* + *σ*/2). As the probability of reentry initiation increases with an increase in fibrosis level, the wavebreaks most often occur in the regions with the highest fibrosis density, which is (*f* + *σ*/2). For reentry initiation this percentage should be larger than 28%; therefore, we expect that for a very large value of *l*, the reentry in heterogeneous tissue will occur if *f* + *σ*/2 ≥ 28%. This line is shown with the dark blue colour in Fig. 2c. We see that our numerical results for *l* = 64 mm (light blue points) are in agreement with this theoretical prediction.

Overall, the phase diagrams demonstrate that a heterogeneous distribution increases the likelihood of arrhythmia onset relative to a homogeneous distribution. Furthermore, they show that the main mechanism of this dependency is the presence of localised tissue patches with larger level of fibrosis.

### Spatiotemporal characteristics of the activation patterns

**Figure 3:**
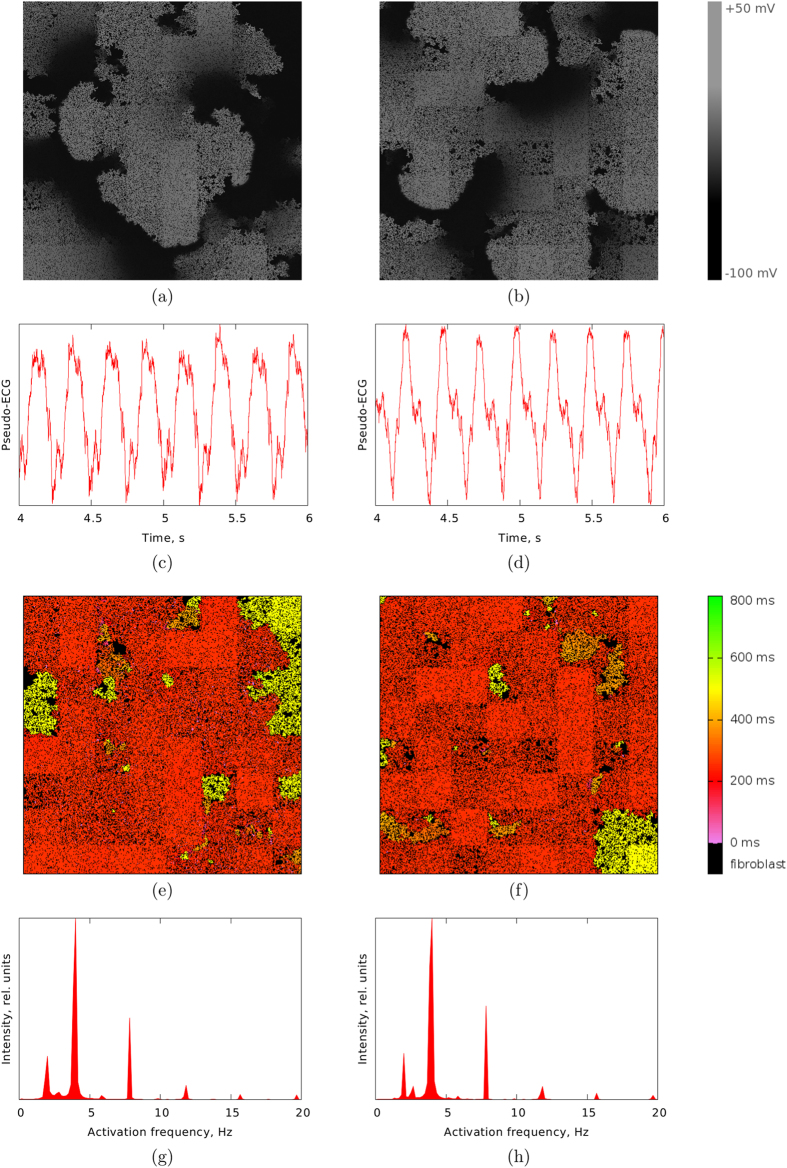
Top (**a**,**b**): two examples of persistent activation patterns emerged in the heterogeneous fibrotic medium by burst pacing: (**a**) arrhythmia mediated with a single independent spiral (Pattern (**a**)); (**b**) multiple wavelets fibrillation (Pattern (**b**)). Middle (**c**,**d**): pseudo-ECG for (**c**) Pattern (**a**) and (**d**) Pattern (**b**). Middle (**e**,**f**): maps of the interbeat interval for (**e**) Pattern (a****) and (**f**) Pattern (**b**). Positions of inexcitable fibroblasts are shown in black. Bottom (**g**,**h**): frequency distibution profiles for (**g**) Pattern (**a**) and (**h**) Pattern (**b**). For these simulations, the values of the parameters are the same as for the simulations presented in Fig. 1: *f* = 25%, *σ* = 25%, *l* = 16 mm.

The induced activation patterns can comprise either only a single spiral (as in Fig. 1) or multiple wavebreaks. Two representative examples of the latter case are demonstrated in the top panel of Fig. 3. We see that the patterns resemble fibrillatory activity. These patterns look fairly similar but, as we will show later, there is a difference between them with respect to the ease of terminating their activity by means of an ablation procedure. We will refer to these representative patterns as Pattern A (Fig. 3a) and Pattern B (Fig. 3b). Pattern A and Pattern B are shown in Supplementary Videos S1 and S2 respectively.

The spatial patterns in Fig. 3a,b look irregular and chaotic. We investigated whether this holds for the overall electrical activity by three approaches. As a first approach, we calculated the ECG generated by these excitation patterns, shown in Fig. 3c,d. As it can be observed from the figure, even though the ECG for both patterns is slightly irregular, the ECG is highly periodic in nature. As a second approach we computed spatial interbeat interval maps produced by the two activation patterns (Fig. 3e,f). In the figure we see that in spite of the heterogeneity in local fibrosis density, the interbeat intervals distribution is largely homogeneous across space, except for a few regions where the interbeat interval is twice as long as in the adjacent areas. This type of spatial interbeat interval distribution is similar to the frequency domains reported during ventricular and atrial fibrillation^27^,^28^ and indicate regions of 1:2 Wenckebach block^29^. Finally, as a third approach we computed mean distribution profile of the activation frequency, shown in Fig. 3g,h. As we can see, the two profiles look almost identically. The dominant frequency peak is at about 4 Hz with a smaller peak at about 2 Hz corresponding to Wenckebach period doubling. This Wenckebach doubling is responsible for the 1:2 component that can be observed in the ECG signal. Based on these results, we conclude that the activation patterns are highly periodic. Similar observations have been reported in experiments with cell cocultures of myocytes and fibroblasts^18^,^30^.

Because of the periodicity of the resulting patterns, it was possible to measure the period and investigate its dependency on the fibrosis distribution.

#### Period of the activation patterns

**Figure 4:**
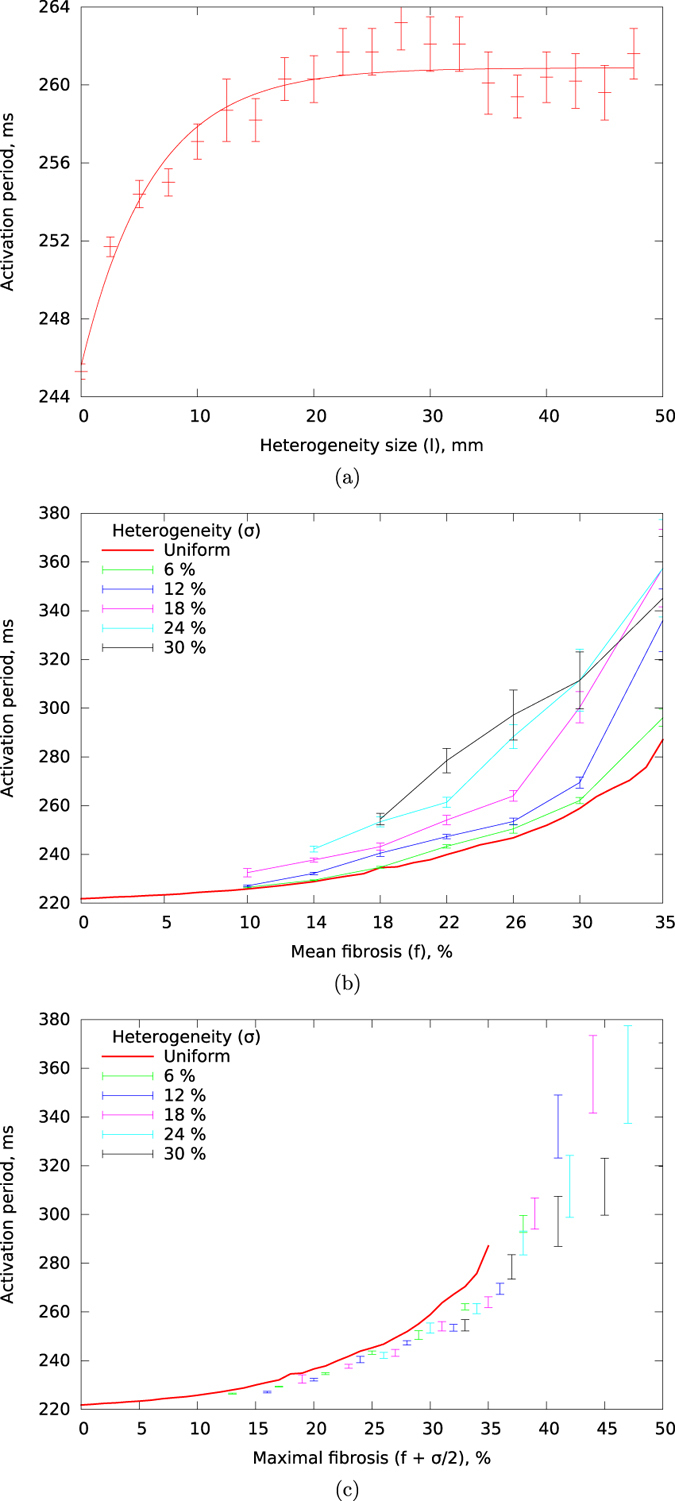
Period of the activation pattern depending on the fibrosis distribution: (**a**) dependence of the period on the size of heterogeneity *l* for *f* = 25% and *σ* = 18%; (**b**) dependence of the period on the mean fibrosis *f* for different values of heterogeneity *σ* and *l* = 40 mm; (**c**) dependence of the period on the value of the maximum local fibrosis level for different values of heterogeneity (*f* + *σ*/2) for *l* = 40 mm.

We investigated how the period of the activation patterns depends on the amount and distribution of fibrosis. The dependency of the period on the size of heterogeneity for a constant mean fibrosis level of *f* = 25% is given in Fig. 4a. We see that period increases with the increase of *l* and saturates for *l* ≥ 20 mm. Similar to our earlier results, the excitation period for the small sized heterogeneity is the same as for homogeneous fibrosis with the same average level. To study what determines the saturated plateau level, we investigated how the period for a constant sized heterogeneity of *l* = 40 mm depends on the extent of heterogeneity *σ* and the average fibrosis level *f*. These results are given in Fig. 4b. For the uniform fibrosis distribution (the red line), we see that period increases superlinearly with the increase of the average fibrosis level *f*, so the rotation of the spiral waves is slowed down in the presence of fibrosis. This is consistent the earlier studies with^14^,^18^. We also see that for heterogeneous fibrosis patterns, the increase in period with average fibrosis levels becomes stronger as the extent of heterogeneity in fibrosis levels increases.

As the shapes of the graphs obtained for different heterogeneity values look similar to each other, we tried to characterise this similarity formally. We found that the graphs will reasonably well match one another if there are all shifted horizontally by *σ*/2 (Fig. 4c). This shift means that to obtain the same period of activation for homogeneous fibrosis as for fibrosis with a heterogeneity of *σ*, we need to use an average fibrosis level of (*f* + *σ*/2) for the homogeneous case versus an average fibrosis level of *f* for the heterogeneous case. As (*f* + *σ*/2) corresponds to the maximal level of fibrosis in heterogeneous tissue, we can conclude that the period for heterogeneous fibrosis is mostly determined by the maximal local fibrosis level, or in other words, by the region where the spiral rotation is slowest.

### Underlying mechanism of the activation patterns

As we showed, despite irregular appearing of spatiotemporal wave patterns, the electrical activation patterns are highly periodic and regular. As the tissue is heterogeneous, these findings may indicate that fibrillation is driven by a single periodic source of excitation, and that the arrhythmia is of the mother rotor type^31^. In the case of mother rotor fibrillation, it is assumed that all wavelets originate from the interaction of a single persistent high frequency source with tissue heterogeneity^31^. To prove that a pattern is of the mother rotor type, one needs to find and remove the mother rotor and demonstrate that fibrillation terminates. This is rather difficult to achieve in experiments but can be done easily in numerical simulations by applying highly targeted virtual ablation of cardiomyocytes.

To classify the type of the activation patterns, we first performed “local ablations”. For this we subdivided the 2D tissue into 64 equally sized squares of size 16 × 16 mm. During an individual local ablation experiment, we “removed” one of these squares, similar to performing a clinical point ablation procedure, replacing the active cardiomyocytes in this region of the tissue by passive “dead” cells.

**Figure 5:**
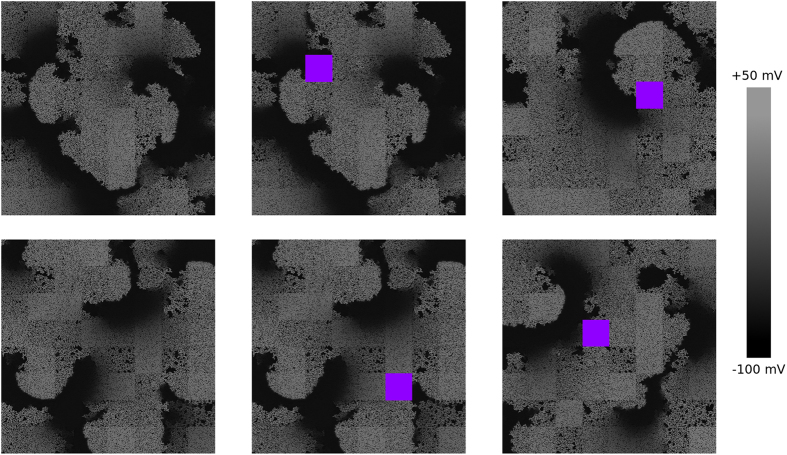
Changes in activation patterns as a result of “square ablation”: removing a small square-shaped part of the tissue. The shade of grey corresponds to the transmembrane voltage. The ablated parts of the tissue are shown in violet. The top row is for Pattern A, and the bottom is for Pattern B. Left column: the activation picture without applying ablation; middle column: ablation performed at a distance from the spiral core; right column: ablation performed at the location of the spiral core.

**Figure 6:**
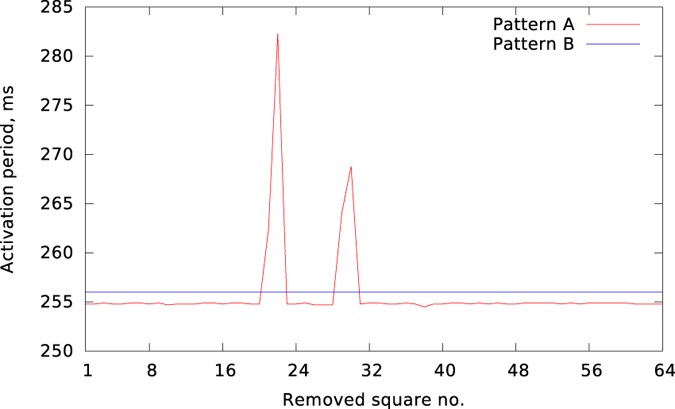
The effect of “square ablation” on the period of Pattern (**A**) and Pattern (**B**). The *x* axis corresponds to the number of the removed part from the 64 possible subdomains.

The results of applying this procedure for our representative examples are demonstrated in Fig. 5. The top pictures correspond to Pattern A. We see that some ablations do not affect the activation pattern (top middle), maintaining the previous number of waves. However, in other cases (top right), the pattern changes drastically, and reduces to a single spiral wave anchored to the ablation site. For Pattern B (bottom row), similar results were obtained. To represent the results of all 64 possible ablation locations, in Fig. 6, we show the period of rotation after performing each of these ablations. As we can see, for Pattern B (the blue line), the ablations do not affect the period of activation. On the other hand, for Pattern A (the red line), while most ablation sites still do not alter the period, a subset of ablation sites exist that result in a significant increase in period of activation, indicative of a substantial slowing down of wave patterns.

In the case of rotors, small, localised ablations are unlikely to remove them. Indeed to terminate spiral wave rotation it is necessary to connect the core of the rotor to the boundary of the medium^32^, effectively forcing the rotor to drift out of the tissue. Therefore, we also performed “line ablations”, a procedure in which a line-shaped ablations was performed that connected a certain place in the tissue to the nearest boundary. We again performed this procedure for the 64 possible locations corresponding to the centres of the 64 squares we previously used for local point ablations.

**Figure 7:**
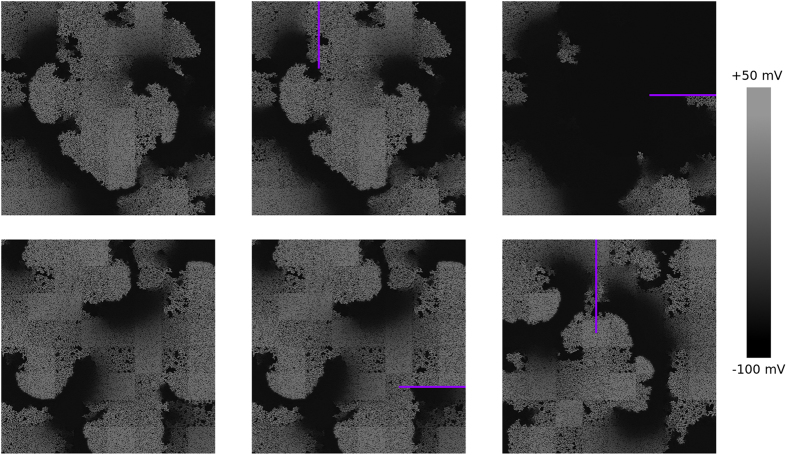
Changes in activation patterns as a result of “line ablation”. Shade of grey corresponds to the transmembrane voltage. Ablations are shown with violet. The top row corresponds to Pattern A and the bottom row to Pattern B. Left column: the activation picture without applying ablation. Middle column: the line ablations where the lines do not go to the cores of the spirals. Right column: line ablations where the lines connect the cores with the boundary.

A few examples of the application of this line ablation procedure are given in Fig. 7. The top row corresponds to Pattern A. We see that there are ablations that do not change the activation pattern (the middle part of the figure). However, for other positions, where the ablation line connected the core of the spiral to a tissue boundary, we observed termination of the arrhythmia (top right part) as a result of the ablation. The three bottom pictures correspond to Pattern B. Here, we never the observed termination of the arrhythmia for any ablation position we chose. These results allow us to conclude that Pattern A is of the mother rotor type and Pattern B is of the multiple wavelet type.

**Figure 8:**
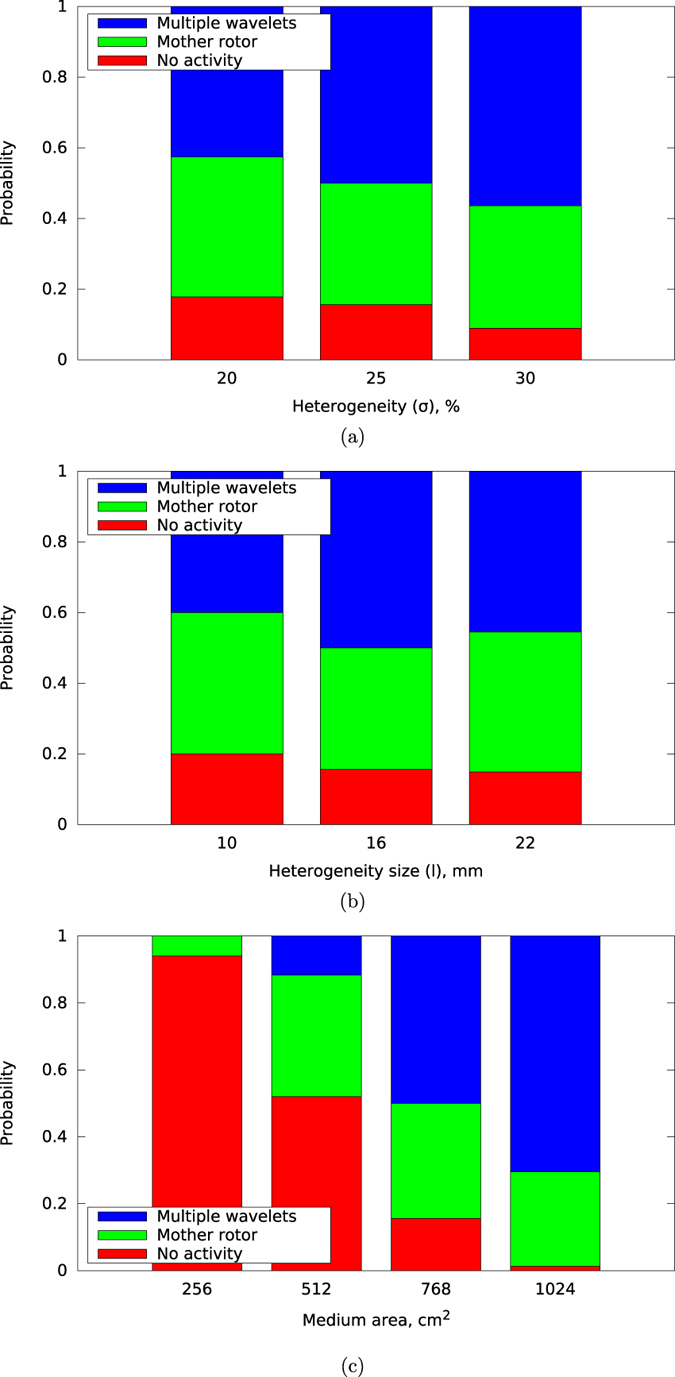
Dependency of the type of the activation pattern on (**a**) the value of heterogeneity, (**b**) the size of heterogeneity, and (**c**) the tissue size. The basic set of the parameter values used for these results is *f* = 25%, *σ* = 25%, *l* = 16 mm, and tissue size of 350 cm^2^. “No activity” means that the burst pacing protocol did not lead to the emergence of persistent activation. The “mother rotor” type of activation pattern was possible to terminate by connecting the core with the boundary. For the “multiple wavelets” type, no cuts of the medium could terminate the arrhythmia.

These methods for determining the type of fibrillation can easily be implemented as an automatic procedure, allowing us to perform a large number of such ablation procedures and subsequent classifications. We used this approach to determine how fibrillation type depends on value of heterogeneity, heterogeneity size, and the tissue size. Figure 8 shows the results of our simulations. In Fig. 8, red indicates absence of induced activity, green indicates mother rotor type fibrillation, and blue indicates multiple wavelet type fibrillation. The sum of the green and blue parts of the column thus indicates the probability of arrhythmia induction. In Fig. 8a, we see that for the larger heterogeneity value the number of cases with multiple wavelet fibrillation increases. The change of the size of heterogeneity within the limits shown in the figure had no significant effect (Fig. 8b). The effect of the tissue size was more pronounced (Fig. 8c). We used values of the tissue size of 40 cm^2^, 150 cm^2^, 350 cm^2^, and 625 cm^2^. For a tissue size of 150 cm^2^ the probability of arrhythmia induction is approximately 50%, and from the blue-green ratio it follows that for the subset of cases that arrhythmia induction occurs 80% of them corresponds to mother rotor type fibrillation. For a smaller tissue of 40 cm^2^ we see that the probability of arrhythmia induction is significantly lower. In addition, it is no longer possible to induce multiple wavelet type fibrillation. If instead we consider larger sized tissues we observe the reverse phenomenon, with the probability of arrhythmia induction increasing and a larger fraction of induced fibrillation being of the multiple wavelet type. For example, for a tissue of 625 cm^2^, arrhythmia induction occurs in almost all cases, and in 75% of these cases it will be of the multiple wavelet type.

Note, that while the larger tissue sizes studied here appear to lie outside the physiologically relevant domain (that is, the size of the human heart), we did not include tissue anisotropy in our current simulations. Adding anisotropy, that is, slower propagation in the transversal direction, is equivalent to an decrease in effective tissue size^33^.

Considering a typical propagation velocity anisotropy of 1:3, an isotropic tissue of size 10 cm × 15 cm = 150 cm^2^, corresponds to an anisotropic tissue of size 10 cm × 5 cm = 50 cm^2^. On a similar note the 350 cm^2^ re-scale to 116 cm^2^ and the 625 cm^2^ to 207 cm^2^. We estimate the surface area of the left human ventricle to be around 160 cm^2^ (see Appendix A in Suppementary Information).

## Discussion

Fibrosis is an important risk factor for arrhythmogenesis since it affects propagation of the excitation wave. It was shown that the fibrosis texture, that is, the layout of fibroblasts, is a more important factor for the onset of arrhythmias than an average level. In this work, we investigated the contribution of the heterogeneity in the distribution of fibrosis in cardiac tissue on arrhythmia formation. We have shown that the heterogeneity of fibrosis promotes the onset of arrhythmias. This effect depends on both the spatial size and the degree of the heterogeneity: a larger size and larger degree of heterogeneity make formation of arrhythmias more probable.

The results in Fig. 2a illustrate that for very small spatial heterogeneity, arrythmogenecity is the same as for homogeneous fibrotic tissue with the same average fibrosis density. Therefore, the degree of heterogeneity is not relevant for this case. For very large spatial heterogeneity arrhythmogenic potential is the same as for homogeneous fibrotic tissue with a fibrosis density equal to the maximum fibrosis density occurring in the heterogeneous tissue. Thus, for larger size of heterogeneity, not the heterogeneity itself but rather the patch with maximum fibrosis density is relevant for the arrhythmogenic potential of the fibrotic tissue. Since, for these two cases, the probability of arrhythmia induction is determined solely by local fibrosis, we assume that it is also true for the intermediate sizes of spatial heterogeneity. This assumption, taken together with the central limit theorem, allows us to explain the hyperbolic shape of the boundary in the *σ*—*l* parametric space (Fig. 2b). A detailed derivation is given in Appendix B in Suppementary Information.

A more detailed study revealed that not only the inducibility of arrhythmia, but also period of activation is determined by the highest level of local fibrosis. This result demonstrates that the arrhythmia sources tend to lie in the regions with the highest fibrosis level. This correlation potentially allows to predict the position of the mother rotor for ablation therapy. A similar correlation between the positions of the rotors and the fibrosis layout has been reported in^28^,^34^. The mechanism responsible for this effect requires additional study.

Another result of our study is the characterisation of the nature of fibrillatory arrhythmias occurring in our model of heterogeneous fibrotic tissue. We found that while spatiotemporal wave patterns look irregular the electrical activity patterns of the arrhythmias have a quite regular, periodic structure. This result indicates that fibrillation is not caused by the type of dynamical instabilities that occur due to a steep restitution curve^35^,^36^. This is in line with the earlier results^14^, where it was shown that the slope of the restitution curve is lower for the values of period of spiral rotation that correspond to fibrotic tissue.

We found that in our case two types of fibrillation are possible: mother rotor and multiple wavelets. These two fibrillation types require different ablation protocols for termination of activity. For the case of multiple wavelets, in addition to normal ablation, one should consider either subdividing the tissue into smaller domains where only the mother rotor fibrillation type is possible (as shown in Fig. 8c) or trying to ablate the rotors by multiple ablation lines.

The mother rotor activation patterns that we obtained are different from the fast mother rotors that were observed in^37^. In Fig. 3e, where the spatial distribution of activation period is given, we do not see any higher frequency regions at the position where the mother rotor is located. Whereas in^37^, the position of the mother rotor correlates with the maximal local activation frequency. In Fig. 3e, where the spatial distribution of the activation period is given, we do not see any higher frequency regions at the position where the mother rotor is located.

The strong periodicity of the activation patterns may indicate the presence of a mother rotor^28^,^38^. We indeed found that for tissue sizes less than 150 cm^2^ (which corresponds to 50 cm^2^ in the anisotropic case), most of arrhythmias were of the mother rotor type (Fig. 8). Even for the tissue as large as 625 cm^2^ (≈208 cm^2^ in the anisotropic case) we still observe mother rotor type fibrillation in 30% of the cases. Interestingly, the multiple wavelet type of fibrillation never occurred for small tissues of 40 cm^2^ (≈13 cm^2^ in the anisotropic case). Therefore, in most realistic cases, one would expect fibrillation to be of the mother rotor type. This may explain why ablations of rotors may remove atrial fibrillation^39^. However, it is still not clear why ablational targeting only the rotor core along is considered effective. In our model, only ablations that connect the spiral core with the boundary of the tissue could effectively terminate the arrhythmia.

In the current study, arrhythmias were induced by a burst pacing protocol. Another commonly used method of triggering arrhythmia is the S1S2 protocol. Our choice for the burst pacing was mainly because this way it is possible to see if arrhythmia can be induced for a particular fibrosis layout. In contrast, the S1S2 protocol always leads to arrhythmia initiation; fibrosis may only contribute to generation of additional excitation sources which may be due to a mechanism that is different from the mechanism of formation of the initial source. Nevertheless, the S1S2 protocol is required for studies targeting the transition from tachycardia to fibrillation which will be addressed in the subsequent research.

Although the probability of the onset of arrhythmias is certainly affected by the stimulation frequency, we expect that this dependency does not significantly change the main results of our paper. Indeed, the results regarding the period of the arrhythmia and its nature were studied after pacing was stopped and should, therefore, not be affected by the initiation procedure. Clearly, as it is known that higher pacing frequencies independently lead to an increase in the likelihood of arrhythmia induction, an increase in the frequency of burst pacing is expected to shift the boundaries on the phase diagrams in Fig. 2 downwards. We can also estimate the lower limit for the leftmost point of the graph presented in Fig. 2a for any value of the pacing period by the following way. As it was shown in^40^,^41^,^42^, to generate a rotor at a geometrical obstacle one needs to pace the tissue with a period that is shorter than the period of spiral wave rotation in that tissue. Therefore, the leftmost point in Fig. 2a should be higher than the level of fibrosis that corresponds to the spiral rotation period equal to the given pacing period. In our case, the pacing period of 240 ms corresponds to the period of spiral wave rotation in a homogeneous tissue with a mean fibrosis level of 22%. This number is smaller than 28% of fibrosis level that is necessary for having reentry in 25% of the cases.

As it is the case for any modelling study, our model represents a simplified description of fibrotic cardiac tissue and as such it has several limitations. A first major model simplifications is to consider solely the effects of structural remodelling. Under many disease conditions the structural remodelling is accompanied by electrical remodelling. Often, the degree of fibrosis is in correlation with the degree of electrical remodelling as well as the extent of ionic heterogeneity. Both electrical remodelling *per se* and ionic heterogeneity are additional factors enhancing the drift of spiral waves^43^,^44^ and the onset of wavebreaks and arrhythmias^45^,^46^. Therefore, we expect that adding electrical remodelling to our model would facilitate arrhythmogenesis.

A second important limitation in the current model is the assumption that myocytes and fibroblasts are not electrically coupled, while some experimental studies indicate that this under some conditions this coupling be the case^47^. However, the issue of such coupling is controversial, with some researchers implying that it exists only in *in vitro*, while others assume that this coupling occurs in cardiac tissue and is facilitated by tissue remodelling. The effect of this coupling requires additional study, outside the scope of our current research. However, we can suggest that in accordance with experiments by^18^ this coupling promotes arrhythmogenesis, therefore, it would probably shift the boundaries on the phase diagrams in Fig. 2 downwards.

It should be noted, however, that while the above model simplifications can be interpreted as too strong, they, in fact, are crucial to allow us to study the effect of fibrosis as obstacles to wave propagation in isolation, without other tissue remodelling factors or other characteristics of fibrosis itself obscuring the results.

A third limitation was that we considered only one type of fibrosis: diffuse fibrosis. Other types of fibrosis are: compact, interstitial, and patchy^5^. It has been reported that patchy fibrosis causes more problems for wave propagation than diffuse fibrosis. The same is valid for interstitial fibrosis if one considers propagation across the fibre direction^5^. On the other hand, compact fibrosis is considered to be the least arrhythmogenic type. Therefore we expect that both patchy and interstitial fibrosis would facilitate the induction of fibrillation, while for compact fibrosis, it might be not possible to induce the arrhythmia in our model at all. Apart from this, we still expect a strong correlation between both the probability of arrhythmogenesis and the period of activation with local fibrosis densities for patchy and interstitial fibrosis. However, to prove these hypotheses an additional study is required.

Heterogeneity in distribution of diffuse fibrosis was modelled by introduction of rectangular patches with different levels of local fibrosis. The square shape is not physiologically based, however, we assume that a particular shape of the fibrotic region has a little impact on arrhythmogenesis.

A fourth limitation was the absence of anisotropy in our model. There are evidences that fibrosis results in increase of anisotropy ratio for a tissue^48^,^49^. Such increase may contribute to breakup formation as it was demonstrated for cell cultures and in simulations studies^50^. Therefore, we expect that the increase of tissue anisotropy due to fibrosis potentially facilitates arrhythmia triggering.

Finally, taking into account the 3D nature of ventricular cardiac tissue would allow for additional 3D mechanisms of spiral breakup that were previously shown to occur in relation to reduced excitability and fibrosis^15^. On the other hand, for 3D tissue, the connectivity is better than for 2D tissue, therefore breakups that potentially occur in 3D are less crucial for the onset of arrhythmia than for 2D. This way we cannot make reliable predictions how the results regarding arrhythmogenesis would change in 3D.

In addition, we assumed that the fibroblasts and the myocytes are similar in size. In real tissue, the shape and the size of a fibroblast is a debate point. Although, an isolated fibroblast is a rounded cell with a diameter of 7–9 *μ*m, a fibroblasts *in vivo* form membrane extensions, which increase its size and surface area^47^. In cell cultures, the fibroblasts tend to flatten more than the cardiomyocytes, effectively increasing their 2D size. A more detailed approach for generation the tissue texture can be found in^51^. That approach attempts to take both the size and anisotropy into account. There are also more advanced models and methods that incorporate realistic anatomical features with superimposing of fibrosis distribution^21^,^22^,^52^. In this study, we used the most straightforward way: the size of a node that can be occupied either by a fibroblast or a myocyte was 0.25 cm. Although this way we neglect finer details of tissue architecture, an indisputable advantage of this approach is its computational efficiency.

Overall, we expect that improving upon our model simplifications will mostly affect our outcomes in a quantitative manner, shifting the ratios between mother rotor versus multiple wavelet dynamics, or shifting the phase diagram boundaries separating reentry from non-reentry. We are confident that our qualitative results that heterogeneity of fibrosis is an additional proarrhythmic factor and that this proarrhythmicity stems mostly from the maximum local fibrosis densities occurring within the heterogeneous tissue, are also valid under alternative model settings.

## Additional Information

**How to cite this article**: Kazbanov, I. V. *et al.* Effects of Heterogeneous Diffuse Fibrosis on Arrhythmia Dynamics and Mechanism. *Sci. Rep.* **6**, 20835; doi: 10.1038/srep20835 (2016).

## Supplementary Material

Supplementary Information

Supplementary Video S1

Supplementary Video S2
