# Supplementary material for: Effects of Heterogeneous Diffuse Fibrosis on Arrhythmia Dynamics and Mechanism
Source: Sci Rep. 2016 Feb 10;6:20835. doi: 10.1038/srep20835 (PMC4748409; doi:10.1038/srep20835)
Supplement: Supplementary Information [file srep20835-s1.pdf]

Effects of Heterogeneous Diffuse Fibrosis on Arrhythmia Dynamics and Mechanism: Supplementary Information

Ivan V Kazbanov[1,*], Kirsten HWJ ten Tusscher[2], and Alexander V Panfilov[1,3]

[1]Department of Physics and Astronomy, Ghent University, Ghent, Belgium
[2]Department of Biology, Utrecht University, Utrecht, the Netherlands
[3]Moscow Institute of Physics and Technology (State University), Dolgoprudny, Moscow Region, Russia
[*]ivan.kazbanov@ugent.be

ABSTRACT

Here, we present Supplementary Information for our manuscript.

Appendix A

Here, we estimate the surface of the human left ventricle. According to,[1] the left human ventricle can be approximated as a part of prolate spheroid with the polar radius of $c \approx 7$ cm, the equatorial radius $a \approx 3$ cm, and the height (apex to base length) $h \approx 10$ cm. The surface area of this body can be computed as a surface of revolution about the z-axis:

$$S = 2\pi \int_{-c}^{h-c} r(z)\sqrt{1+(r'(z))^2}dz, \tag{S1}$$

where the radius $r(z)$ is:

$$r(z) = a\sqrt{1-\left(\frac{z}{c}\right)^2}. \tag{S2}$$

Thus, the integral is:

$$S = 2\pi a \int_{-c}^{h-c} \sqrt{1-\frac{c^2-a^2}{c^2}\left(\frac{z}{c}\right)^2}dz. \tag{S3}$$

Using the eccentricity $e = \sqrt{c^2-a^2}/c$ and denoting $l = h-c$, we can rewrite the integral as:

$$S = 2\pi a \int_{-c}^{l} \sqrt{1-\left(\frac{ez}{c}\right)^2}dz = \frac{2\pi ac}{e}\int_{-e}^{le/c} \sqrt{1-\xi^2}d\xi. \tag{S4}$$

Since

$$\int \sqrt{1-\xi^2}d\xi = \frac{1}{2}\left(\arcsin\xi + \xi\sqrt{1-\xi^2}\right) + C, \tag{S5}$$

we obtain:

$$S = \frac{\pi ac}{e}\left(\arcsin\frac{le}{c} + \frac{le}{c}\sqrt{1-\left(\frac{le}{c}\right)^2} + \arcsin e + e\sqrt{1-e^2}\right). \tag{S6}$$

Substituting the numbers, we get:

$$e \approx 0.90 \tag{S7}$$

$$\frac{le}{c} \approx 0.39 \tag{S8}$$

$$S \approx 165\ \text{cm}^2. \tag{S9}$$

Appendix B

Here, we illustrate that the hyperbolic dependency of the onset of arrhythmia on the size of the heterogeneity (the boundary in Figure 2b) directly follows from the assumption that the only parameter that determines the probability of spiral wave formation is the local level of fibrosis f_{loc}. Indeed, if we define the local fibrosis level f_{loc} as an average fibrosis in an area of a certain characteristic length a (a-region), then the number of heterogeneous "small squares" of the size l occupying this area is: $n \sim (a/l)^2$. The probability distribution of fibrosis in an a-region, in accordance with the central limit theorem, is given by the Gaussian function:

$$w(f_{\mathrm{loc}}) = \frac{1}{\sqrt{2\pi}\Sigma} \exp\left(-\frac{(f_{\mathrm{loc}} - f)^2}{2\Sigma^2}\right), \tag{S10}$$

where Σ is the root mean square of the distribution of fibrosis in an a-region. The distribution of fibrosis in each of the n "small squares" has the variance $\sigma_{\mathrm{sq}}^2 \sim \sigma^2$, where σ is the parameter that we introduced to quantify the degree of heterogeneity. According to the central limit theorem Σ can be estimated as:

$$\Sigma \approx \sqrt{\frac{\sigma_{\mathrm{sq}}^2}{n}} \sim \sqrt{\frac{\sigma^2}{n}} \sim \sqrt{\frac{\sigma^2}{(a/l)^2}} \sim \frac{\sigma l}{a}. \tag{S11}$$

Thus, we see that the distribution $w(f_{\mathrm{loc}})$ does not change if σl is constant. According to our assumption that the probability of arrhythmia induction depends only on the local level of fibrosis, it follows that if $\sigma l = \mathrm{const}$, then the probability of arrhythmia induction also stays constant. Since $\sigma l = \mathrm{const}$ is an equation for a hyperbola, this explains the hyperbolic shape of the boundary in the σ—l parameter space (Figure 2b).

References

1. Streeter, D. D. J. Gross morphology and fiber geometry of the heart. *Handbook of Physiology* **1**, 61–112 (1979).

Supplementary Video Captions

Supplementary Video 1. Pattern A: an example of a persistent activation pattern of the mother rotor type induced in the tissue with heterogeneous fibrosis distribution by burst pacing.

Supplementary Video 2. Pattern B: an example of a persistent activation pattern of the multiple wavelets type induced in the tissue with heterogeneous fibrosis distribution by burst pacing.
